# Is complete surgical resection of stage 4 neuroblastoma a prerequisite for optimal survival or may >95 % tumour resection suffice?

**DOI:** 10.1007/s00383-012-3109-3

**Published:** 2012-06-22

**Authors:** S. Zwaveling, G. A. M. Tytgat, D. C. van der Zee, M. H. W. A. Wijnen, H. A. Heij

**Affiliations:** 1 Department of Paediatric Surgery, Wilhelmina Children’s Hospital, University Medical Centre Utrecht, PO Box 85090, 3508 Utrecht, The Netherlands; 2 Paediatric Surgical Centre of Amsterdam, Academic Medical Centre and VU University Medical Centre, Amsterdam, The Netherlands; 3 Department of Paediatric Oncology, Emma Children’s Hospital, Academic Medical Centre Amsterdam, Amsterdam, The Netherlands; 4 Department of Paediatric Surgery, Radboud University Nijmegen Medical Centre, Nijmegen, The Netherlands

**Keywords:** Neuroblastoma, Stage 4, Surgery, Resection, Survival

## Abstract

Numerous studies have shown that for optimal survival in localized International Neuroblastoma Staging System stage 1–3 neuroblastoma, complete tumour resection (CR, macroscopic total tumour removal) is usually mandatory. In contrast, it is conceivable that in stage 4 disseminated disease, less extensive surgery [gross total resection (GTR), >95 % tumour removal] may suffice. This review shows substantial survival benefit in studies reporting on stage 4 patients undergoing CR, but also in studies reporting on patients undergoing GTR. Comparison between these studies is severely hampered by treatment heterogeneity. We found only four studies that explicitly compared survival between patients undergoing either CR or GTR. Two of these studies showed favourable results for patients treated with CR, while the other two did not show differences in survival.

## Introduction

Over the years, the contribution of surgery towards survival of International Neuroblastoma Staging System (INSS) stage 1, 2 and 3 patients has become apparent [1–6]. Since tumours in these stages are still localized, it is comprehensible that this approach may lead to optimal survival. In contrast, the optimal level of surgery in stage 4 (disseminated) tumours is subject of discussion. Due to the metastatic nature of this stage some experts postulate that local control by means of complete resection may not improve survival—or may even be detrimental—and less extensive surgery, combined with other therapies, may suffice. In publications focussing on this matter, survival following a certain level of excision is usually compared with survival following all less radical surgery, often including biopsies. This obviously blurs the interpretation of these observations. It seems more logical to compare a true radical resection with a narrowly irradical resection, a situation resembling the actual situation in the OR more closely. In order to explore this matter more deeply, we particularly searched for manuscripts comparing survival after true complete resection [gross complete resection (CR), total macroscopic tumour removal] with survival after moderately less extensive surgery [gross total resection (GTR), >95 % tumour removal].

## Methods

PubMed was searched using MeSH heading Major Topic “neuroblastoma” with subheading “surgery” AND (stage IV OR stage 4). This was compared with the following set of search terms: Neuroblastoma AND (surgery OR surgical OR resection OR excision) AND (stage IV OR stage 4 OR high-risk) AND (survival OR mortality). Field: title/abstract. Article in English. The latter search strategy provided a wider palette of publications, encompassing the publications retrieved with the former search strategy, and was used in this review.

We found 172 articles. The title and abstracts were thoroughly screened and papers focussing on the influence of the extent of surgery on survival in stage 4 neuroblastoma were selected and studied in detail. Only manuscripts that explicitly described the degree of surgical resection were considered eligible. Finally, 20 articles were included.

## Surgery for neuroblastoma

### INSS and surgical terminology

Since the INSS was introduced in 1988 a complete gross resection (CR), as distinct from incomplete resection (IC), is defined as the macroscopic total removal of all visible tumour and nearby abnormal lymph nodes. The presence or absence of residual microscopic tumour is not counted in the terms of complete gross resection [7]. Because complete gross excision is often difficult to obtain, some authors added the term near-complete excision [8]. This is defined as excision of the tumour leaving a minimal macroscopic residue. Others have proposed an additional subdivision of excisions, namely gross total resection (GTR; removal of more than 95 % of the visible tumour), subtotal resection (STR; removal of more than 50 % but less than 95 %) and less than STR (removal of less than 50 %) and a biopsy [9, 10]. This multitude of surgical terms was recently discussed by Kubota [11] and is summarized in Table 1. In this manuscript we use the INSS definition for CR (total macroscopic tumour removal), while GTR is defined as >95 % removal of all macroscopic tumour. The extent of the resections in the reviewed articles was judged accordingly and terminology was adjusted if necessary.

**Table 1:** Terminology related to the degree of the surgical resection

Level of resection	Resectability
Complete gross resection (CR)	Macroscopic total removal of all visible tumour and nearby abnormal lymph nodes
Near-complete gross resection	Resection of tumour leaving a minimal macroscopic residue
Gross total resection (GTR)	Removal of >95 % of the visible tumour
Incomplete resection	
Subtotal resection (STR)	Removal of >50 % but <95 % of the visible tumour
Less than STR	Removal of <50 % of the visible tumour

### INSS stage 1–3

The important role of radical surgery in the treatment of localized stage 1–3 neuroblastoma is nowadays widely acknowledged [1–6] and will not be further addressed in this review.

### INSS stage 4

Several reports aimed at determining the optimal level of surgery in stage 4 patients have been published. Of the 20 studies reviewed in this article, in 16 complete resection was attempted (Table 2a, 3rd column), whereas in four the authors tried to achieve gross total excision (Table 2b, 3rd column). In ten of these studies the authors eventually consider themselves in favour of GTR, while in ten CR is advocated as the procedure of choice (Table 2a, b, 6th column).

**Table 2:** Studies reporting on operated stage 4 patients in whom either complete gross resection (CR) or gross total resection (GTR) was attempted

Publication	Number of operated patients	Intended level of surgery (achieved percentage between brackets)	Improved survival compared to all less extensive surgery	Improved survival of CR explicitly compared to GTR	Supported type of surgery
a. Complete gross resection (CR)					
Haase et al. [3]	52	CR^a^	Yes (S)	ND	CR
Chamberlain et al. [15]	28	CR (46 %)	Yes (S)	ND	CR
Kuroda et al. [16]	24	CR^a^	Yes (S)	ND	CR
Browne et al. [30]	30	CR (63 %)	Yes (NS)	ND	GTR
Sultan et al. [20]	291	CR (58 %)	Yes (S)	ND	CR
Escobar et al. [21]	104	CR (50 %)	Yes (S)	ND	CR
La Quaglia et al. [18]	70	CR^b^ (56 %)	Yes (S)	ND	CR
La Quaglia et al. [19]	141	CR^b^ (73 %)	Yes (S)	ND	CR
Adkins et al. [14]	468	CR (45 %)	Yes (NS)	**Yes (NS)**	CR
Koh et al. [17]	26	CR (38.5 %)	Yes (S)	**Yes (S)**	CR
Shorter et al. [26]		CR	No	**No**	GTR
Castel et al. [9]	71	CR (55 %)	No	**No**	GTR
von Allmen et al. [27]	69	CR^a,b^	No	ND	GTR
von Schweinitz et al. [6]	791	CR (59 %)	No	ND	GTR
Kiely et al. [25]	75	CR (75 %)	No	ND	GTR
Le Tourneau et al. [12]	36	CR (33 %)	No	ND	CR
b. Gross total resection (GTR)					
Kaneko et al. [24]	10	GTR (70 %)	Yes (NS)	ND	GTR
Matsumura et al. [28]	214	GTR (64 %)	Yes (NS)	ND	GTR
Tsuchida et al. [29]	92	GTR (79 %)	Yes (S)	ND	GTR
McGregor et al. [10]	107	GTR (77 %)	No	ND	GTR

In the 4th column, the survival of patients undergoing the intended level of surgery is compared to patients undergoing all less extensive surgery. In the 5th column, if applicable (indicated by bold letter type), survival after CR is compared to GTR

*ND* not determined, *S* significant, *NS* not significant

^a^Percentage cannot be determined

^b^CR according to INSS, the term GTR is used in the publication

#### CR versus GTR

In most studies either CR or GTR was compared to patients undergoing all less extensive surgery. Because this may have resulted in the inclusion of patients with substantial tumour remnants, bias may occur. Only four studies we found explicitly compared survival after CR to survival after GTR (Table 2a, 5th column). In one of those studies a significant survival benefit for CR over GTR was demonstrated. Another study also showed a survival benefit for CR, but this was not significant. The other two studies showed no differences in survival following CR or GTR.

#### Studies favouring CR

Based on a series of 36 operated patients Le Tourneau et al. postulated that CR in stage 4 patients may contribute to prolonged survival, although survival did not significantly differ from patients undergoing less extensive surgery. The authors stress that metastatic control is more important than the level of surgery and is probably essential for optimal survival [12]. Haase reported a significant survival and disease free survival benefit 3 years after CR compared to less extensive surgery, but survival percentages were not separately mentioned in this manuscript [13]. In a large study by the same group, Adkins et al. found that complete resection was possible in 27 %. This increased to 45 % when surgery was preceded by chemotherapy. CR led to significantly improved 5-year event-free survival when compared to less extensive surgery (26 vs. 19 %) [14]. Chamberlain studied 28 patients and concluded that complete surgical resection resulted in a clear survival benefit over less radical surgery. The 3-year survival was reported to be 40 and 15 %, respectively [15]. A Japanese study described a series of patients—twenty-four at stage 4 and nine at stage 3. Total excision was defined as excision free of macroscopic residual tumour and can be considered identical to CR according to the INSS. Almost 52 % of the patients undergoing CR reached a disease free survival of over 5 years. The four patients with residual disease all died [16]. These results have to be interpreted with caution because they are derived from both stage 3 and stage 4 patients. From another study of 26 patients, it was concluded that complete surgical resection, defined by the authors as the absence of microscopic residual disease, leads to significant survival benefit. CR was achieved in 10 children (38.5 %). In this group the 5-year survival rate was 65 % compared with 0 % in the group with residual tumour mass [17]. La Quaglia [18] demonstrated a clear survival benefit in 39 patients undergoing CR in 1994. Notably, in this study actually the term GTR was used. GTR was defined as removal of all visible and palpable tumour from the primary site and regional lymphatics. This is the same definition used by the INSS for complete resection [7] and therefore the level of surgery was considered to be CR in this review. In a more recent study by the same team the survival benefit in patients undergoing CR was reconfirmed. CR was accomplished in 73 % and led to increased overall survival. Fifteen years after surgery 50 % of patients treated with CR was still alive, compared to 10 % of the patients who did not undergo CR [19]. In an article analysing 291 stage 4 patients, Sultan demonstrated that CR could be achieved in 58 %. The 5-year survival estimate was 53.2 % compared to 35.7 % in patients who did not undergo CR (significant difference) [20]. A study by Escobar of 104 operated patients also favours CR, showing significant survival benefit [21]. In two other publications CR was advocated as well, but survival data were not explicitly reported [22, 23].

#### Studies favouring GTR

Other studies show no improvement in survival following CR when compared to less extensive surgery [6, 9, 24–27] (Table 2a, 4th column). Based on data from the late seventies and early eighties, Shorter concluded that CR is not a prerequisite for optimal treatment of stage 4 neuroblastoma [26]. Following incomplete resection, survival rates were even better than after CR with a 4-year survival of 45 and 15 %, respectively. The authors hypothesized that the biological characteristics of the tumour, rendering them favourable or unfavourable, were far more important prognostic markers than the extent of the resection. Likewise, based on a small series, Kiely [25] suggested that incomplete excision may be enough and that more extensive surgery does not necessarily prolong survival. From a series of 71 procedures, Castel reported no differences in event-free 5-year survival between patients undergoing various levels of resection. The only significant difference in survival was found when these patients were compared to patients solely undergoing biopsy [9]. Event-free 5-year survival rates were 0 % for children having biopsy only, 25 % for resection of less than 50 % tumour mass, 31 % for 50–90 % resection, 44 % for >90 % resection and 33 % for complete resection. The authors conclude that the final outcome is determined more by metastatic relapses than by the degree of resection. von Allmen et al. show similar results. In that study the term GTR was used, but this was identical to the definition of complete resection used by the INSS. Seventy-six patients underwent surgery. Seven patients had stage 3 neuroblastoma and 69 suffered from stage 4 disease. Unfortunately, the reported survival was pooled for both groups. The authors were able to achieve CR in 63 % of the patients, while >90 % resection could be achieved in 16 % and 50–90 % resection was achieved in 8 %. Local recurrence occurred in patients undergoing less extensive surgery, but not in patients undergoing CR. Event-free and overall survival (60 %) were not influenced by the extent of resection [27]. von Schweinitz showed that CR was a prerequisite for prolonged event-free survival in localized disease (stages 1–3). In contrast, survival in stage 4 patients did not diminish after incomplete resection compared with CR [6]. Here too, the authors conclude that incomplete resection may be feasible and that risky complications can be avoided.

These results support the vision of authors who consider GTR to be an adequate level of surgery for stage 4 tumours (Table 2b). In a large study Matsumura reported that removal of approximately 100 % of the tumour (which can be interpreted as GTR by INSS standards) can only lead to a slightly improved survival (not significant) when compared with less radical resection. The authors found resolution of metastases to be far more important [28]. In addition, the Study Group of Japan for Treatment of Advanced Neuroblastoma concluded that GTR (defined as >98 % removal of tumour in that study) resulted in lower local recurrence rate and better survival than partial resection. Two-year survival was 59 and 47 %, respectively [29]. A publication by Browne, primarily aimed at accomplishing CR, showed a lower metastatic recurrence rate following CR compared to incomplete resection and better survival (68 vs. 55 %, respectively), but this was not significant and may have been caused by differences in tumour biology or undetected micrometastasis in the bone marrow. No differences in local control were found. The authors state that aggressive surgical resection, including removal of organs, is no longer necessary due to improved medical therapies [30]. Likewise, Kaneko postulated that highly extensive surgery may not be required due to better pre-and postoperative chemotherapy protocols [24]. In ten operated patients the 5-year overall and relapse-free survival was 56 and 46 %, respectively. In all but one no CR was performed. Instead, seven of the patients underwent GTR, while in two gross macroscopic residual was left. Although not enough patients undergoing CR were available to make a comparison with GTR in this study, it indicates that substantial survival can be achieved if tumour mass is left behind after surgery. Contrary to these positive results, we found one study that demonstrated no difference in survival following GTR or following less extensive surgery [10].

## Discussion

The optimal extent of surgery in INSS stage 4 patients is still a matter of debate. Since tumours in this stage are no longer localized, some have postulated that local control by complete resection may not be necessary for optimal survival and less extensive surgery (GTR) may suffice. In ten of the 20 studies included in this review, the authors eventually seem in favour of GTR, while the other ten articles advocate CR as the procedure of choice, clearly depicting the present lack of consensus (Table 2a, b, 6th column). Indeed, supporters of both CR and GTR claim good results. Nonetheless it is should be noted that in this review in 50 % of the articles focussing on CR a significant improvement in survival compared with less extensive surgery was shown (Table 2a, 4th column). In the papers focussing on GTR this was only reported in 25 %, but the number of articles in the GTR group is obviously too small to draw conclusions (Table 2b, 4th column). Still, the possibility that this means that CR may be advantageous over GTR has to be acknowledged. On the other hand, a substantial number (38 %) of papers describing the effects of CR on survival reports no improvement compared to less extensive surgery at all [6, 9, 25–27] (Table 2a, 4th column). Of the four publications that explicitly make a comparison between CR and GTR [9, 14, 17, 26] a significant survival benefit (for CR) was shown in one case only (Table 2a, 5th column) [17]. Therefore, at present no definite conclusions can be drawn on the optimal extent of surgery.

This inconsistency can partly be attributed to the lack of a universal tumour staging system and to the diversity in surgical terminology before the INSS was introduced in 1988 [7]. The analysis and comparison of literature published before this date is therefore challenging. But even articles using the INSS are difficult to interpret. With the introduction of the International Neuroblastoma Risk Group (INRG) Classification system in 2009, finally a consensus approach for pre-treatment risk stratification has been developed [31] and this will make future comparison less complicated. Currently, most studies are retrospective and in many cases patient numbers are small, making reported statistical differences occasionally questionable. In several studies the results may have been contaminated by pooling patients with INSS stage 3 and 4 tumours and determining survival rates for these groups as a whole [13, 16, 27, 32]. Moreover, in spite of the adoption of the INSS, the definition of CR is still not always correctly applied [18, 19, 27] and the proposed subdivision of the extent of surgery [9, 10]—including GTR—is not universally used. In some of the studies that supposedly do seem to use the terms GTR or CR correctly, the actual level of excision is not described, so inaccuracies cannot be excluded. In addition, the comparison of literature is hampered by the fact that treatment for stage 3 and 4 neuroblastoma is multidisciplinary. Various and continuously changing chemotherapy and irradiation regiments are used and this may obscure the effects of surgery on overall survival. Surgeon-dependent factors, such as experience and skills, and the chosen surgical approach are presumably also of importance. The timing of the surgery itself, which varies considerably between studies, could influence survival as well. Moreover, it cannot be excluded that selection bias has occurred in the comparative studies that we reviewed. It is possible that CR was attempted in patients with a better overall condition and that, in an effort to reduce the risks of surgery, patients in a more incapacitated state underwent GTR or incomplete resection. Furthermore, in the majority of the studies that compare various levels of surgery only two groups are studied: patients undergoing the most extensive type of surgery and the remainder of the patients undergoing less aggressive surgery. The second group is obviously very heterogeneous and could contain patients with abundant tumour load. As a consequence, the favourable results of the group undergoing extensive surgery may be relatively exaggerated. Finally, the presence or absence of unfavourable tumour characteristics could obscure the relevance of the surgical procedures. Due to variation in histological and biological features the prognosis of seemingly comparable tumours, both in mass and in growth, can differ dramatically. In addition, these tumour characteristics may influence the operative method that is chosen, thus resulting in selection bias. In this respect it is for instance conceivable, as suggested by von Schweinitz [6], that GTR may be suitable for stage 4 disease if MYCN is not amplified but that complete excision may be beneficial in case of MYCN amplification. However, important prognostic data, such as the MYCN amplification and the Shimada histology [33, 34] were either not determined or not incorporated in the results of most studies.

The question whether survival is determined more by recurrence of locoregional disease or by recurrent metastases cannot be answered definitely due to the lack of relevant data on this subject. However, several authors do report that the final outcome of high-risk neuroblastoma patients is more related to the evolution of metastases than to the extent of resection [9, 27, 28, 30]. Because it is conceivable that, compared to GTR, CR may not only lead to higher peri-operative morbidity and mortality, but also to more late complications (such as renal failure or chronic diarrhoea) [25] this is an important issue to address. Interestingly, a recent large study suggests that the degree of resection may not be related to the rate of complications. The complication rate in high-risk patients was reported to be 29, 38 and 36 % in complete resections, minimal residual resections and partial resections, respectively [14]. Nevertheless, it is comprehensible that GTR is technically easier to accomplish than CR. If GTR can be considered an adequate level of resection in stage 4 disease, this could lead to an increase in the percentage of successfully performed operations. In fact, one study showed that even after initial radiation therapy in 66 % of the patients a GTR could be achieved, whereas in patients not treated with radiation GTR was possible in 89 % [35]. This matches with the observation that in the manuscripts reviewed here the percentages in which GTR was successfully obtained tend to be higher than the percentages in which CR could be accomplished (mean 73 and 54 %, respectively, Table 2a, b, 3rd column).

We conclude that, based on the heterogeneity in treatment modalities and differences in diagnostics, a true comparison of the effect of surgery on survival between the present studies on this matter is severely hampered. Only four publications explicitly make a comparison between CR and GTR, while keeping other variables stable. A significant survival benefit (for CR) was shown in one case only. The comparison of the other studies in this review should be interpreted with caution. It is obvious that additional research is necessary. With the development of uniformly applied treatment protocols the opportunity to study the effects of surgery, independently of other factors, has finally emerged. In 2010 in The Netherlands, the Dutch Childhood Oncology Group (DCOG) NBL 2009 Treatment Protocol, which is based on the GPOH (Gesellschaft für Pädiatrische Onkologie und Hämatologie) NB2004 High-risk Protocol, was adopted. Imaging techniques are becoming increasingly important in staging of neuroblastoma [36]. In the DCOG HR neuroblastoma patients, we will perform preoperative imaging by MRI and digital postoperative photographs of the operation field. In this way, all image defined risk factors can be determined and the digital photographs will be reviewed by a board of surgeons to definitively define the ultimate level of resection. This will be done in a prospective study, so we will hopefully be able to compare outcome of surgery with preoperative IRDS and biological characteristics such as MYCN-status, histology and ploidy.

## References

[1] De Bernardi B, Mosseri V, Rubie H (2008). Treatment of localised resectable neuroblastoma. Results of the LNESG1 study by the SIOP Europe Neuroblastoma Group. Br J Cancer.

[2] Perez CA, Matthay KK, Atkinson JB (2000). Biologic variables in the outcome of stages I and II neuroblastoma treated with surgery as primary therapy: a children’s cancer group study. J Clin Oncol.

[3] Haase GM, Atkinson JB, Stram DO, Lukens JN, Matthay KK (1995). Surgical management and outcome of locoregional neuroblastoma: comparison of the Childrens Cancer Group and the international staging systems. J Pediatr Surg.

[4] Modak S, Kushner BH, LaQuaglia MP, Kramer K, Cheung NK (2009). Management and outcome of stage 3 neuroblastoma. Eur J Cancer.

[5] Powis MR, Imeson JD, Holmes SJ (1996). The effect of complete excision on stage III neuroblastoma: a report of the European Neuroblastoma Study Group. J Pediatr Surg.

[6] von Schweinitz D, Hero B, Berthold F (2002). The impact of surgical radicality on outcome in childhood neuroblastoma. Eur J Pediatr Surg.

[7] Brodeur GM, Seeger RC, Barrett A (1988). International criteria for diagnosis, staging, and response to treatment in patients with neuroblastoma. J Clin Oncol.

[8] Cecchetto G, Mosseri V, De BB (2005). Surgical risk factors in primary surgery for localized neuroblastoma: the LNESG1 study of the European International Society of Pediatric Oncology Neuroblastoma Group. J Clin Oncol.

[9] Castel V, Tovar JA, Costa E (2002). The role of surgery in stage IV neuroblastoma. J Pediatr Surg.

[10] McGregor LM, Rao BN, Davidoff AM (2005). The impact of early resection of primary neuroblastoma on the survival of children older than 1 year of age with stage 4 disease: the St. Jude Children’s Research Hospital Experience. Cancer.

[11] Kubota M (2010). The role of surgery in the treatment of neuroblastoma. Surg Today.

[12] Le Tourneau JN, Bernard JL, Hendren WH, Carcassonne M (1985). Evaluation of the role of surgery in 130 patients with neuroblastoma. J Pediatr Surg.

[13] Haase GM, O’Leary MC, Ramsay NK (1991). Aggressive surgery combined with intensive chemotherapy improves survival in poor-risk neuroblastoma. J Pediatr Surg.

[14] Adkins ES, Sawin R, Gerbing RB, London WB, Matthay KK, Haase GM (2004). Efficacy of complete resection for high-risk neuroblastoma: a Children’s Cancer Group study. J Pediatr Surg.

[15] Chamberlain RS, Quinones R, Dinndorf P, Movassaghi N, Goodstein M, Newman K (1995). Complete surgical resection combined with aggressive adjuvant chemotherapy and bone marrow transplantation prolongs survival in children with advanced neuroblastoma. Ann Surg Oncol.

[16] Kuroda T, Saeki M, Honna T, Masaki H, Tsunematsu Y (2003). Clinical significance of intensive surgery with intraoperative radiation for advanced neuroblastoma: does it really make sense?. J Pediatr Surg.

[17] Koh CC, Sheu JC, Liang DC, Chen SH, Liu HC (2005). Complete surgical resection plus chemotherapy prolongs survival in children with stage 4 neuroblastoma. Pediatr Surg Int.

[18] La Quaglia MP, Kushner BH, Heller G, Bonilla MA, Lindsley KL, Cheung NK (1994). Stage 4 neuroblastoma diagnosed at more than 1 year of age: gross total resection and clinical outcome. J Pediatr Surg.

[19] La Quaglia MP, Kushner BH, Su W (2004). The impact of gross total resection on local control and survival in high-risk neuroblastoma. J Pediatr Surg.

[20] Sultan I, Ghandour K, Al-Jumaily U, Hashem S, Rodriguez-Galindo C (2009). Local control of the primary tumour in metastatic neuroblastoma. Eur J Cancer.

[21] Escobar MA, Grosfeld JL, Powell RL (2006). Long-term outcomes in patients with stage IV neuroblastoma. J Pediatr Surg.

[22] Cantos MF, Gerstle JT, Irwin MS (2006). Surgical challenges associated with intensive treatment protocols for high-risk neuroblastoma. J Pediatr Surg.

[23] Hase T, Ohta S, Tani T (2002). Outcome of infants with neuroblastoma detected by mass screening and surgically treated in Shiga Prefecture, Japan: what is the role of surgery?. Pediatr Surg Int.

[24] Kaneko M, Ohakawa H, Iwakawa M (1997). Is extensive surgery required for treatment of advanced neuroblastoma?. J Pediatr Surg.

[25] Kiely EM (1994). The surgical challenge of neuroblastoma. J Pediatr Surg.

[26] Shorter NA, Davidoff AM, Evans AE, Ross AJ, Zeigler MM, O’Neill JA (1995). The role of surgery in the management of stage IV neuroblastoma: a single institution study. Med Pediatr Oncol.

[27] von Allmen D, Grupp S, Diller L (2005). Aggressive surgical therapy and radiotherapy for patients with high-risk neuroblastoma treated with rapid sequence tandem transplant. J Pediatr Surg.

[28] Matsumura M, Atkinson JB, Hays DM (1988). An evaluation of the role of surgery in metastatic neuroblastoma. J Pediatr Surg.

[29] Tsuchida Y, Yokoyama J, Kaneko M (1992). Therapeutic significance of surgery in advanced neuroblastoma: a report from the study group of Japan. J Pediatr Surg.

[30] Browne M, Kletzel M, Cohn SL, Seshadri R, Reynolds M (2006). Excellent local tumor control regardless of extent of surgical resection after treatment on the Chicago Pilot II protocol for neuroblastoma. J Pediatr Surg.

[31] Cohn SL, Pearson AD, London WB (2009). The International Neuroblastoma Risk Group (INRG) classification system: an INRG Task Force report. J Clin Oncol.

[32] Moon SB, Park KW, Jung SE, Youn WJ (2009). Neuroblastoma: treatment outcome after incomplete resection of primary tumors. Pediatr Surg Int.

[33] Maris JM (2005). The biologic basis for neuroblastoma heterogeneity and risk stratification. Curr Opin Pediatr.

[34] Weinstein JL, Katzenstein HM, Cohn SL (2003). Advances in the diagnosis and treatment of neuroblastoma. Oncologist.

[35] Robbins JR, Krasin MJ, Pai Panandiker AS (2010). Radiation therapy as part of local control of metastatic neuroblastoma: the St. Jude Children’s Research Hospital experience. J Pediatr Surg.

[36] Brisse HJ, McCarville MB, Granata C (2011). Guidelines for imaging and staging of neuroblastic tumors: consensus report from the international neuroblastoma risk group project. Radiology.

